# José Carlos Mariátegui a la luz del prisma de la historia de la discapacidad

**DOI:** 10.1590/S0104-59702024000100044

**Published:** 2024-10-11

**Authors:** Carolina Ferrante

**Affiliations:** i Investigadora adjunta, Consejo Nacional de Investigaciones Científicas y Técnicas, Departamento de Ciencias Sociales/Universidad Nacional de Quilmes. Ciudad Autónoma de Buenos Aires − Argentina caferrante@gmail.com

José Carlos Mariátegui es apreciado como “el intelectual peruano más importante del siglo XX” (Drinot, 2023, p.11). Nacido en 1894 y fallecido en 1930, devendría un protagonista central del pensamiento nacional y popular latinoamericano al ser considerado “el primer marxista” de la región. La envergadura de su rol, lo ha convertido en extenso objeto de estudio historiográfico. No obstante, a través del libro *José Carlos Mariátegui o el “cojito genial”*, Paulo Drinot analiza la figura de Mariátegui focalizando en un aspecto hasta ahora pasado por alto como ha sido la cuestión de su discapacidad.

Mariátegui, a los 7 años, tiene un accidente que le generaría una cojera permanente y que desencadenaría una trayectoria vital atravesada por la discapacidad y las atenciones médicas. Siendo un niño, pasaría un largo tiempo hospitalizado en la Maison de Santé, una clínica de la Sociedad Francesa de Beneficencia, ubicada en Lima. Posterior a ello, y a lo largo de toda su vida, diversos malestares le ocasionarían itinerarios terapéuticos que tendrían un punto de inflexión en 1924, cuando su pierna derecha es amputada. Así, como señala Drinot (2023, p.12), en la vida de Mariátegui: “La discapacidad se convierte tanto en una lucha diaria como en un elemento constitutivo de su identidad y un reto personal”. Sin embargo, llamativamente, en los estudios históricos dedicados al intelectual peruano, esta condición es un elemento no problematizado, bien sea por la ausencia de un análisis crítico respecto a la discapacidad, o por la negación de su existencia.

Partiendo de este vacío, Drinot recupera los aportes de la *disability history* para analizar la figura de Mariátegui. Desde esta corriente, que surge a principios de este siglo en el mundo anglosajón, como emergente del campo más amplio de los *disability studies* y que cuenta con escasos antecedentes a nivel latinoamericano, recupera la premisa de comprender a eso, llamado contemporáneamente discapacidad, como una construcción social y cultural (Brégain, 2022; Ferrante, Ramacciotti, 2021). Desde esta perspectiva, las respuestas sociales brindadas a eso, catalogado como “cojera” o “discapacidad”, están mediadas por representaciones configuradas socio históricamente en un contexto acotado y ante las cuales los/as agentes poseen diversos grados de incorporación o cuestionamiento. Así, Drinot utiliza el prisma de la historia de la discapacidad para volver a mirar a la figura de Mariátegui y plantear dos interrogantes: (1) ¿cómo se ha representado socialmente la discapacidad de Mariátegui a través de imágenes y de textos escritos?; (2) ¿qué impacto tuvo en su protagonista la experiencia de la discapacidad? Para dar respuesta a estas preguntas, Drinot recurre a diversas fuentes recolectadas en el Archivo José Carlos Mariátegui. Las mismas incluyen numerosas fotografías e imágenes que retratan al protagonista en situaciones de su vida política, familiar y social y correspondencia escrita.

A través del análisis del corpus, Drinot evidencia que socialmente se construiría una retórica acerca de la discapacidad de Mariátegui desde la cual la misma dotaba compensatoriamente a su portador de una genialidad única, una excepcionalidad que le permitía “superar”, lo que en esa época se consideraba como desgracia. Para caracterizar esta representación, Drinot acude a la figura del *supercrip*, propuesta desde el campo de los *disability studies*, para describir una imagen de la persona con discapacidad donde la misma es representada como “digna” a causa de superar extraordinariamente la presunta tragedia que implica la discapacidad. Esta retórica imputa un sentido moralizante a su portador, al configurarse como ejemplo para el resto de las personas portadoras de cuerpos capaces. A través del análisis de la correspondencia de Mariátegui, Drinot estudia cómo, en gran medida, él mismo contribuye a afianzar esta construcción. Asimismo, advierte la exclusión del tema de la discapacidad en la obra de Mariátegui, quien se interesó por otros sectores marginalizados (como la cuestión “indígena” o la “obrera”). El análisis contempla la vida pública, política, familiar y social, contemplando la discapacidad de modo interseccional a otras variables (como, por ejemplo, el género). También considera el papel de las redes afectivas de apoyo en las posibilidades de la significación imputada.

Asimismo, centrándose en la vivencia encarnada de la discapacidad, Drinot reconoce cómo la búsqueda de recuperación y rehabilitación fue un motor central en la vida de Mariátegui. De hecho, la posibilidad de migrar a Buenos Aires, planeada en los últimos años de su vida, tras la amputación de su pierna, posee como principal motivación la intención de acceder a una prótesis con la cual poder volver a caminar. La capital Argentina, por aquellos años, constituía un espacio de referencia en América Latina para el tratamiento de amputaciones y discapacidad motrices. En las décadas de 1920 y 1930, el desarrollo de los saberes médicos en la materia eran muy incipientes, y eso condicionaba seriamente las posibilidades de sobrevida de sus protagonistas.

El texto forma parte de la colección Perú Breve, que busca acercar temas académicos de relevancia social a la ciudadanía general. El texto se compone de una introducción, cinco apartados y una conclusión. La obra posee una lectura muy amigable, sin resignar capacidad analítica.

En el prefacio del texto, Alberto Vergara indica que el libro aquí reseñado “abre caminos”, y considero que esta apreciación es completamente justa. En su afán por “explicar” el fenómeno Mariátegui, el texto brinda una clave analítica novedosa e ilumina un área de enorme vacancia académica en América Latina: la historia de la discapacidad. Drinot recupera plenamente este prisma. Lo hace abordando un tema inédito y revelando un aspecto sin antecedentes hasta ahora a nivel regional: el análisis de caso. Los pocos estudios realizados de la historia de la discapacidad latinoamericana han centrado su atención en fenómenos más amplios, como por ejemplo: la lucha por los derechos de las personas con discapacidad; las respuestas médicas y sociales a la discapacidad; las políticas de Estado (Ferrante, Ramacciotti, 2021; Brégain, 2022). Pero además, Drinot brinda un programa para la investigación en historia de la discapacidad que yo creo que apuesta a una clave latinoamericana. ¿Por qué? Porque parte del reconocimiento de temas relevantes para nuestro espacio social. Así, señala como áreas de vacancia: la reconstrucción de una historia de la discapacidad recuperando la/s cosmovisión/es de los pueblos originarios y contemplando el rol de la colonización en los procesos modernos de significación de la discapacidad. También advierte sobre la importancia de analizar la discapacidad en clave interseccional, contemplando el cruce de esta variable con otras, como, por ejemplo, el género, la edad, la “raza”, la clase social.

Asimismo, como toda buena obra, la lectura del texto abre preguntas para ser abordadas por futuras indagaciones: ¿qué tipo de respuestas médicas y sociales recibían las personas con diversas discapacidades en Perú en el período analizado?; ¿cómo ha sido considerada la figura de Mariátegui por el movimiento de personas con discapacidad peruano?

Por los motivos expuestos, celebro la publicación de este libro, que estoy convencida poseerá ecos amplios en los estudios críticos latinoamericanos en el área de discapacidad (Danel, Pérez Ramírez, Yarza de los Ríos, 2021).
